# Whole exome sequencing identifies an *AMBN* missense mutation causing severe autosomal-dominant amelogenesis imperfecta and dentin disorders

**DOI:** 10.1038/s41368-018-0027-9

**Published:** 2018-09-03

**Authors:** Ting Lu, Meiyi Li, Xiangmin Xu, Jun Xiong, Cheng Huang, Xuelian Zhang, Aiqin Hu, Ling Peng, Decheng Cai, Leitao Zhang, Buling Wu, Fu Xiong

**Affiliations:** 1 Department of Stomatology, Nanfang Hospital, College of Stomatology, Southern Medical University, Guangzhou, Guangdong China; 2 Department of Medical Genetics, School of Basic Medicine Sciences, Southern Medical University, Guangzhou, Guangdong China; 3 Guangdong Key Laboratory of Biological Chip, Guangzhou, Guangdong China; 4 Department of Laboratory Medicine, ZhuJiang Hospital, Southern Medical University, Guangzhou, Guangdong China

## Abstract

Tooth development is a complex process that involves precise and time-dependent orchestration of multiple genetic, molecular, and cellular interactions. Ameloblastin (AMBN, also named “amelin” or “sheathlin”) is the second most abundant enamel matrix protein known to have a key role in amelogenesis. Amelogenesis imperfecta (AI [MIM: 104500]) refers to a genetically and phenotypically heterogeneous group of conditions characterized by inherited developmental enamel defects. The hereditary dentin disorders comprise a variety of autosomal-dominant genetic symptoms characterized by abnormal dentin structure affecting either the primary or both the primary and secondary teeth. The vital role of *Ambn* in amelogenesis has been confirmed experimentally using mouse models. Only two cases have been reported of mutations of *AMBN* associated with non-syndromic human AI. However, no *AMBN* missense mutations have been reported to be associated with both human AI and dentin disorders. We recruited one kindred with autosomal-dominant amelogenesis imperfecta (ADAI) and dentinogenesis imperfecta/dysplasia characterized by generalized severe enamel and dentin defects. Whole exome sequencing of the proband identified a novel heterozygous C-T point mutation at nucleotide position 1069 of the *AMBN* gene, causing a Pro to Ser mutation at the conserved amino acid position 357 of the protein. Exfoliated third molar teeth from the affected family members were found to have enamel and dentin of lower mineral density than control teeth, with thinner and easily fractured enamel, short and thick roots, and pulp obliteration. This study demonstrates, for the first time, that an *AMBN* missense mutation causes non-syndromic human AI and dentin disorders.

## Gene mutation pinpointed in tooth development disorders

A mutation on a gene involved in healthy tooth development may cause both enamel and dentin disorders. The ameloblastin enamel protein, and its associated gene, *AMBN*, play vital roles in enamel formation and tooth remodelling. Mutations on *AMBN* can cause amelogenesis imperfecta (AI), a genetic and hereditory condition resulting in enamel defects and severe tooth decay. Now, Fu Xiong and Bu-Ling Wu at Southern Medical University in Guangzhou, China, and co-workers have identified an *AMBN* mutation found in both enamel and dentin defect disorders. The researchers analyzed extracted teeth from a Chinese patient with both AI and a severe dentin disorder, along with teeth from affected and non-affected members of the same family, and compared the results with a control group. They identified a rare mutation on *AMBN* common to all affected individuals.

## Introduction

Ameloblastin (AMBN: MIM *601259) expression was first detected in the enamel organ and is the second most abundant enamel matrix protein, after amelogenin, known to have key roles in enamel formation.^1,2^ The extracellular matrix has a critical role in tissue development and homoeostasis by mediating cell growth, migration, differentiation, apoptosis, and gene expression.^3,4^ In the enamel extracellular matrix, AMBN is believed to participate in ameloblast attachment to the underlying enamel matrix^5^ and in modulation of enamel crystal growth,^6^ and mutations in this gene are already known to cause amelogenesis imperfecta (AI: MIM104530).^7,8^ AI is the term given to a heterogeneous group of disorders characterized by failure of normal amelogenesis, with a reported prevalence of between 1/700 and 1/14 000.^9,10^ Genes associated with non-syndromic AI encode proteins involved in the formation and maintenance of the developing enamel matrix, including amelogenin (*AMELX*), enamelin (*ENAM*), kallikrein-related peptidase 4 (*KLK4*), matrix metallopeptidase 20 (*MMP20*), Golgi-associated secretory pathway pseudokinase (*FAM20A*), chromosome 4 open reading frame 26 (*C4ORF26*), and amelotin (*AMTN*).^8,11–17^ Other genes associated with non-syndromic AI include solute carrier family 24 member 4 (*SLC24A4*) and G-protein-coupled receptor 68 (*GPR68*), which are involved in ion transport^18,19^; laminin subunit beta 3 (*LAMB3*), integrin subunit beta 6 (*ITGB6*), collagen type XVII alpha 1 chain (*COL17A1*), and laminin subunit alpha 3 (*LAMA3*), which are involved in extracellular matrix adhesion^20–25^; family with sequence similarity 83 member H (*FAM83H*) and WD repeat domain 72 (*WDR72*), which are associated with intracellular vesicles^26,27^; acid phosphatase 4 (*ACP4*), which is a hydrolytic enzyme^28,29^; dentin sialophosphoprotein (*DSPP*), which is associated with dentin development^30^; and distal-less homeobox 3 (*DLX3*), which is associated with craniofacial development.^31^ Enamel abnormality is characteristic of mouse models with a deletion of *Ambn* exons 5 and 6 (*Ambn* ^−5,6/−5,6^)^32,33^, and *Ambn* overexpression.^33^ For many years, there were no known mutations of *AMBN* associated with AI phenotypes. Recently, hypoplastic AI was found to be associated with homozygous exon 6 deletion^8^ and another family was identified with a novel homozygous splice-site mutation (c.532–1G > C).^34^ To date, no point mutations of *AMBN* have been found to be responsible for this disease.

Dentinogenesis is a highly ordered process in which the organic predentin matrix is progressively mineralized by ectomesenchymally derived cells called odontoblasts.^35^ The odontoblasts differentiate at the bell stage of tooth development, forming a single layer of cells lining the pulp cavity, where they secrete the organic predentin matrix into the underlying space.^36^ AMBN expression is also detected in pulpal mesenchymal cells, including preodontoblasts and young odontoblasts.^37^ *AMBN* is known to be involved in the mesenchymal–ectodermal interaction that precedes dentin and enamel secretion, and the sequential expression pattern of AMBN also acts as a signalling molecule.^38^ Currently, hereditary dentin disorders are divided into dentinogenesis imperfecta (DGI) and dentin dysplasia (DD).^39^ Recent genetic studies have shown that mutations of collagen type I alpha 1 chain (*COL1A1*), collagen type I alpha 2 chain (*COL1A2*), *DSPP*, secreted modular calcium-binding protein 2 (*SMOC2*), vacuolar protein sorting 4 homologue B (*VPS4B*), and ssu-2 homologue (*SSUH2*) cause dentin disorders.^39–49^ *AMBN* can enhance reparative dentin formation, which confirms that this protein has an important role in odontoblast differentiation and dentinogenesis.^50^ The enamel defects confirm the observations from mouse models and show that human AI is associated with disrupted Ambn function. However, to date, mutations of *AMBN* have not been associated with the phenotypes of hereditary dentin disorders. In this study, we report, for the first time, that *AMBN* mutations cause both non-syndromic human AI and hereditary dentin disorders.

## Results

### Tooth phenotype

**Fig. 1:**
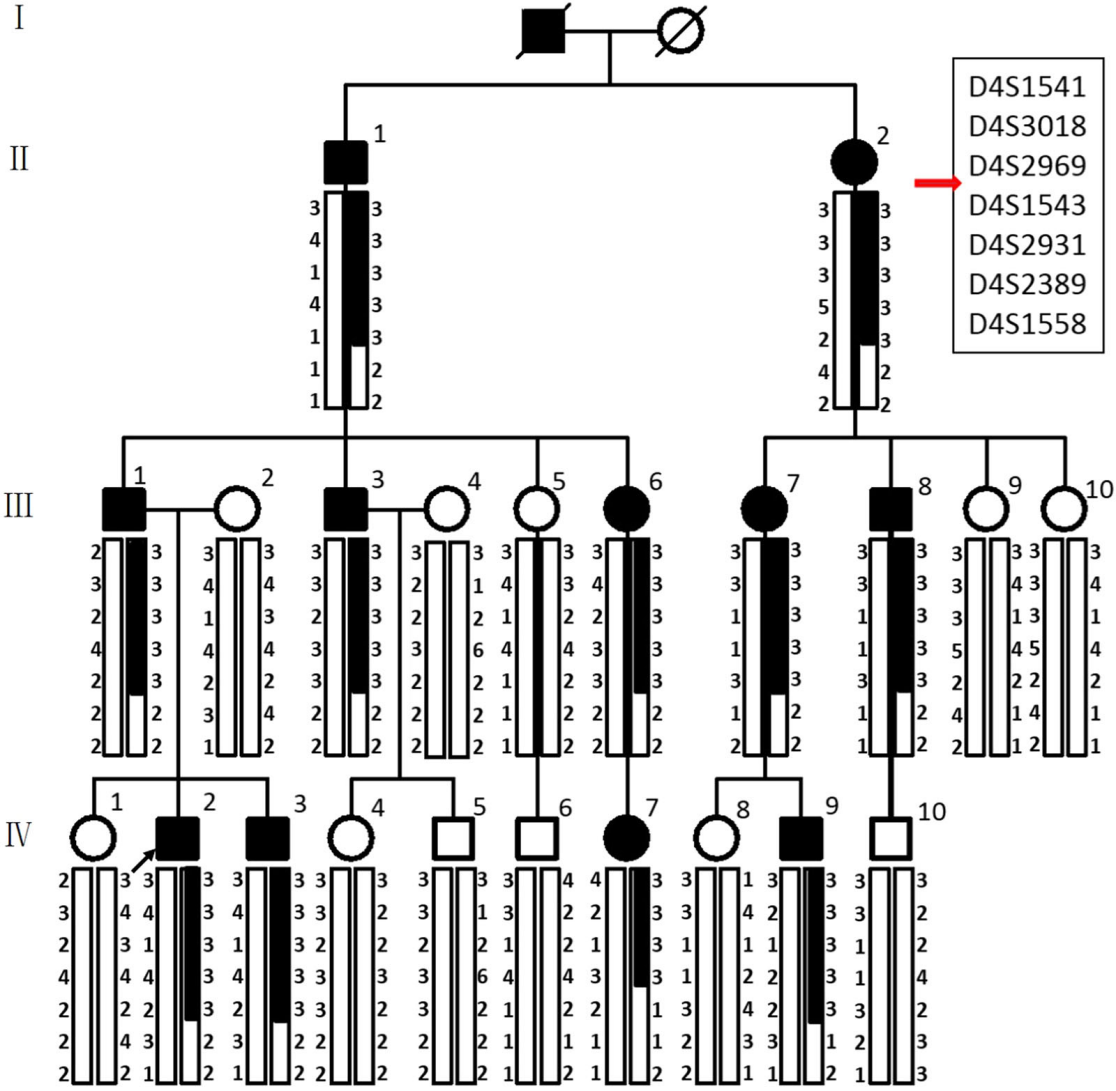
Family pedigree and pedigree-based linkage study of affected and unaffected individuals. The segregating haplotype is indicated by a box with seven polymorphic microsatellite markers on 4q13.1-4q21.1. *AMBN* is flanked by D4S2969 and D4S1543. Affected subjects are denoted in black. The proband is indicated by an arrow.

**Fig. 2:**
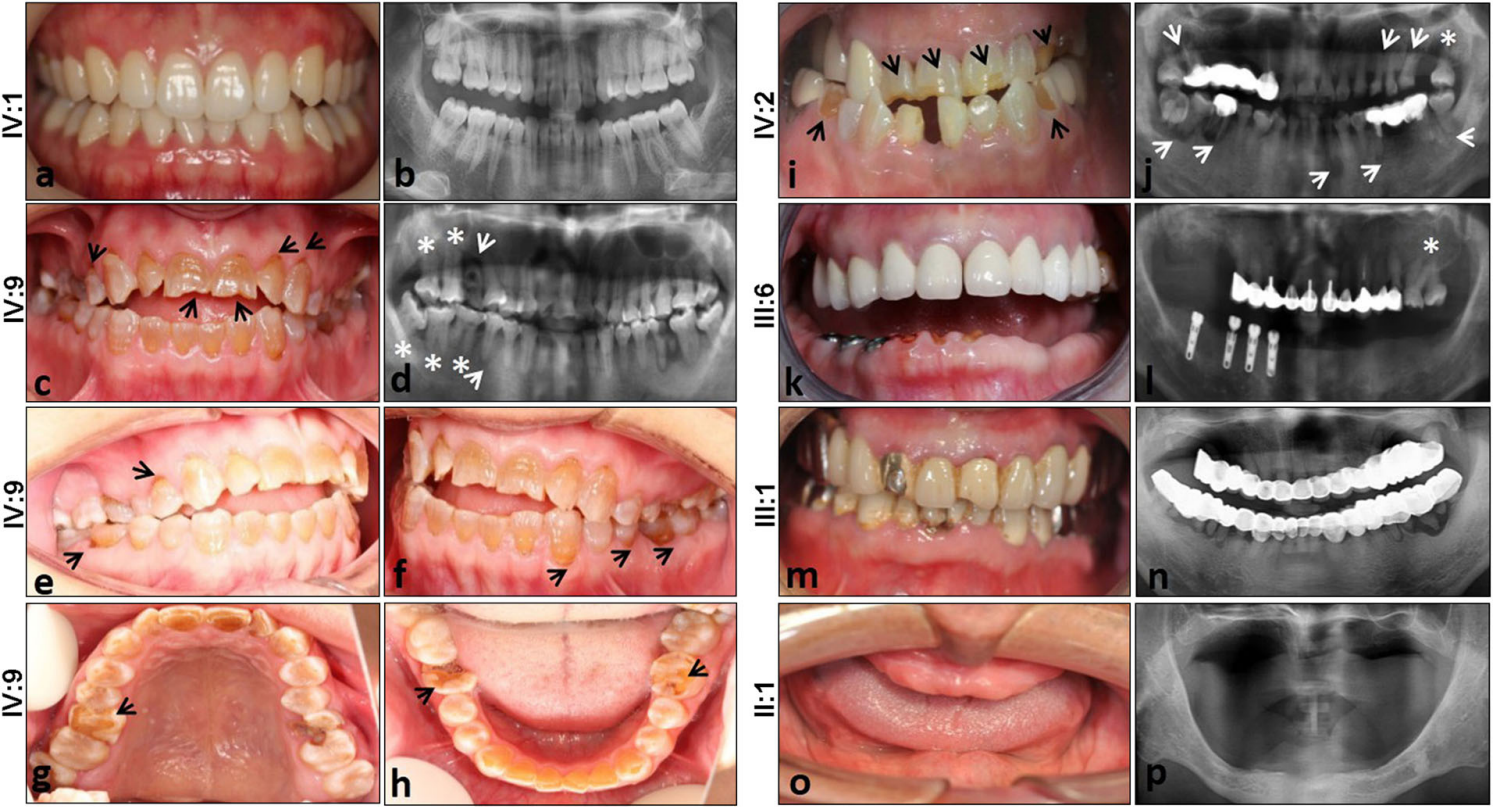
Dental phenotype and panoramic radiograph of the patients. **a** Normal permanent teeth; **b** panoramic radiograph of normal teeth. Dental phenotype of IV:9 **c**,**e**–**h**. An intraoral view of a male patient at the age of 21 shows brown crowns with thin and chipping enamel, especially in the cervical part of all teeth (black arrows), and short crowns of the permanent maxillary central incisors worn out by attrition (black arrow). **d**,**j**,**l** Panoramic radiographs of patients show bulbous crowns covered with thin enamel, cervical constriction (asterisk), short constricted roots, and apical radiolucencies in the second molars (white arrows). **i** An intraoral view of a patient at the age of 22 shows grey crowns with enamel chipped and absent in places. **k**,**m** Above the age of approximately 40, patients had lost almost all of their teeth or had several loose roots. **l**,**n** Patients were treated by long-span fixed partial denture repair, **l** implant prosthodontics, **k** removable partial denture, or **o** removable complete denture according to their family economic situation. Finally, **o**,**p** all patients lost all their teeth at an early age (generally by their 50s).

We identified a Chinese family with generalized enamel and dentin defects involving the primary and secondary dentitions. On clinical examination of the teeth (Fig. 1), 11 individuals (II:1, II:2, III:1, III:3, III:6, III:7, III:8, IV:2, IV:3, IV:7, and IV:9) were affected, exhibiting hypomineralized AI and hereditary dentin disorders, but were in good general health and exhibited no abnormalities upon extraoral examination. Unaffected individuals (III:2, III:5, III:9, III:10, IV:1, IV:4, IV:5, IV:6, IV:8, and IV:10) did not carry the mutation and exhibited normal dentition. The crowns of the affected family members appeared grey or brownish-blue (Fig. 2c, e–h, i). The enamel was thinner and often dislodged because of alterations to the enamel–dentin junction. Consequently, the exposed hypomineralized dentin was quickly worn away by attrition. The radiographic aspect showed bulbous crowns because of an important cervical constriction, short and thick roots, and pulp obliteration with periapical radiolucencies in non-carious teeth (Fig. 2d, j, l). With development of the disease, the absorption of periapical bone gradually increased, which affected the tooth-supporting tissues, leading to loosening of the teeth and eventually early tooth loss. Two teeth of the secondary dentition of individual family member IV:9 were in a high grade of mobility due to circumferential alveolar bone resorption, although this individual was only 21 years old (Fig. 2d). There were six secondary teeth missing in individual IV:2 and four loosened secondary teeth with large-area periapical bone defects in non-carious condition (Fig. 2j), although this individual was 22 years old. All the other family members who were heterozygous for the mutation had similar experiences: detached enamel, circumferential alveolar bone resorption in non-carious teeth, tooth loosening, and eventual tooth loss. By the age of approximately 40, they lost almost all of their teeth or had only a few loose roots remaining (Fig. 2k–n). To maintain the physical occlusion position and improve masticatory function, the patients received a long-span fixed partial denture repair (Fig. 2k–n), removable partial denture (Fig. 2k–i), implant prosthodontics (Fig. 2k-i), or removable complete denture (Fig. 2o,p) according to their family economic situation.

**Fig. 3:**
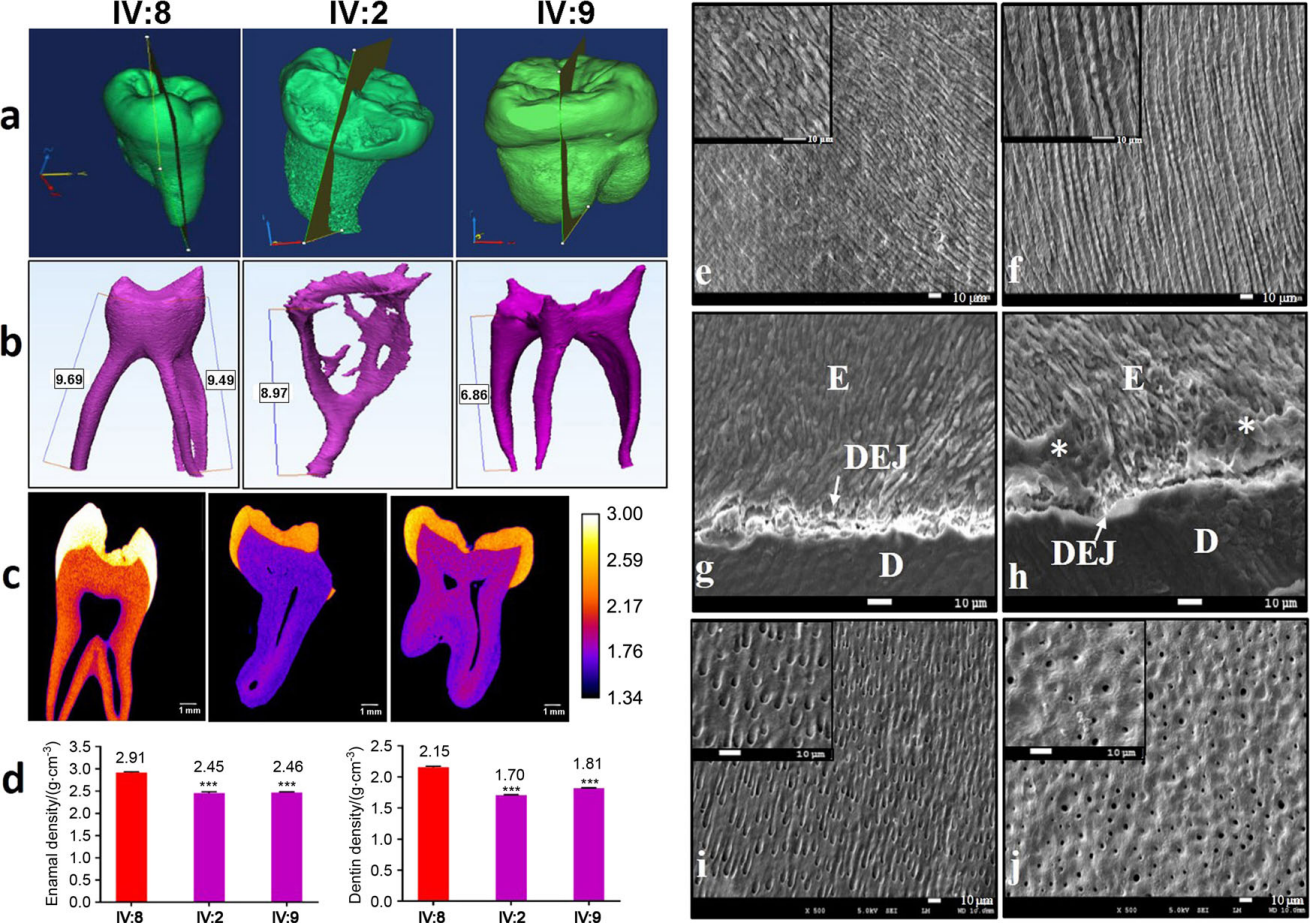
Tooth ultrastructural analyses. **a**–**d** High-resolution X-ray CT analysis of exfoliated teeth from control individual IV:8 and affected individuals IV:2 and IV:9. **a** 3D reconstruction of the tooth CT data: individual IV:8 and individuals IV:2 and IV:9. **b** 3D reconstruction of pulp chambers. Teeth of IV:2 and IV:9 exhibit thistle-shaped pulp chambers. **c** Typical CT sections through the teeth are presented using false colour calibrated with respect to mineral density to generate mineral density maps. Scale bar is marked in g/cm^3^. **d** Mean enamel and dentin mineral density for each tooth is also shown graphically. The control tooth of IV:8 exhibits enamel and dentin apparently normal in structure and density. Affected teeth of IV:2 and IV:9 exhibit enamel and dentin significantly reduced in mineral density compared with the normal tooth (*P* < 0.001). *P < 0.05, **P < 0.01, or ***P < 0.001. Videos of 3D-rendered CT data showing surface detail and the internal structure of all teeth are available as Supplementary Material. **e**–**j** SEM of representative exfoliated teeth. **e** Tooth of individual IV:8 exhibits normal enamel architecture comprising prisms (rods) of individual enamel crystallites. **f** Enamel of patient IV:9 tooth is in the normal range in terms of the order but characterized by fewer enamel prisms (rods) and wider inter-rod distances, **h** with occasional areas exhibiting a disturbed structure (asterisk). **h** The width of the DEJ of patient IV:9 is increased and the shapes of transitions within the DEJ zone are straight instead of sigmoidal. **i** SEM of the control tooth of individual IV:8 shows normal dentin structure. **j** The distribution of dentin tubules was not even and the size of the dentin tubular diameter was not consistent with the tooth of patient IV:9. **j** Most peritubular dentin is thicker and dentin tubes are smaller or obliterated completely compared to the control tooth.

Three extracted disused mandibular third molar teeth from IV:8, IV:2, and IV:9 were available for phenotypic characterization after obtaining their informed consent (the donors were approximately 20–22 years old). High-resolution computed tomography scanning (Fig. 3a–c and Supplemental Videos 1-3) of control tooth 1 from IV:8 and teeth 2 and 3 from IV:2 and IV:9 showed that tooth 1 exhibited a normal enamel layer in terms of thickness and mineral density (enamel density = (2.91 ± 0.06) g·cm^‒3^ compared with a range of (2.57–3.10) g·cm^‒3^ previously reported for enamel)^51–55^ and a normal dentin mineral density, whereas teeth 2 and 3 exhibited reduced enamel and dentin mineral density (Fig. 3c–d). Enamel thickness also appeared to be reduced. The pulp chambers were narrower with pulp chamber calcification (Fig. 3b). The roots were shorter, with small or obliterated root canals (Fig. 3b).

When examined with scanning electron microscopy (SEM), the normal control teeth exhibited the prismatic structure characteristic of normal human enamel and dentin (Fig. 3e, g, i). SEM revealed that the mutation had little effect on the prismatic structure, but the numbers of enamel prisms of affected teeth were obviously reduced (Fig. 3f); the enamel also displayed distinct regions where the enamel structure was particularly disturbed (Fig. 3h). The outline of the dentin–enamel junction (DEJ) was changed: the width of the DEJ was increased and the structure of sigmoidal transitions within the DEJ zone, which firmly combine enamel and dentin together, became a straight line in appearance (Fig. 3h). Examination of the ultrastructure of dentin of affected teeth showed that the distribution of dentin tubules was not even and that the dentin tubular diameter was not consistent (Fig. 3j). This verified that there was an increase in the thickness of major peritubular dentin in mutant teeth, which reduced the size of or even obliterated the dentin tubules compared to control teeth (Fig. 3j).

### Genetic analysis

**Fig. 4:**
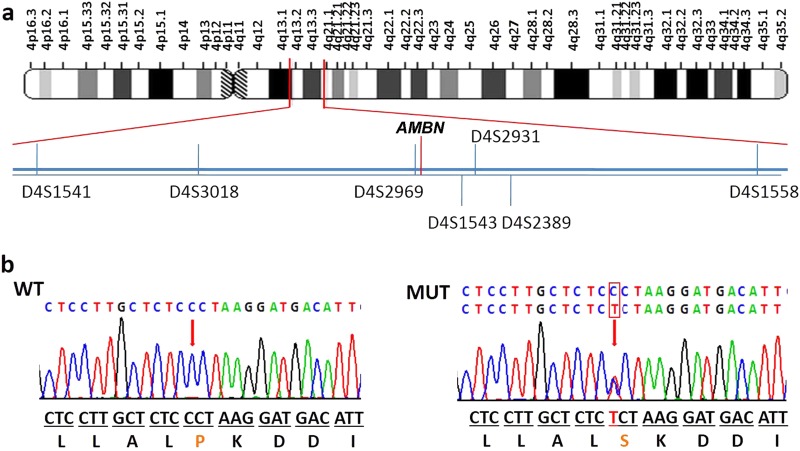
Interval mapping and mutational analysis of *AMBN*. **a** Chromosomal location of the *AMBN* gene and the distribution of seven short tandem repeat (STR) markers. **b** Sanger sequencing result of the WT and the mutant. All the patients in this family carry a heterozygous mutation of *AMBN* (c.1069C > T). **b** This abnormal variation changes amino acid 357 of AMBN from a hydrophobic P to a hydrophilic S.

To identify the pathogenic variant responsible for the phenotype, we first screened for mutations in the coding and splice regions of the *DSPP* gene (exons 1–5). However, no mutations were identified. To confirm this result, long-range PCR was conducted with primers designed to amplify all exons, including the highly repetitive sequence in exon 5 of *DSPP*. Sequencing of the resulting product revealed that there were no mutations present within the *DSPP* exons or the flanking splice regions. In addition, we also analysed the copy number variations (CNVs) in the *DSPP* gene via array comparative genomic hybridization. Data analysis revealed no pathogenic CNVs in the *DSPP* gene in affected individuals in this family. Next, we performed exome sequencing with genomic DNA for affected individuals IV:2 and IV:9, and control IV:1 in collaboration with Genesky-Shanghai (China). Sequence analysis of all exons showed that 47 potentially pathogenic variants were shared by the two affected individuals and not by the unaffected individual. None of these variants were within genes known to be involved in AI, except for *AMBN*, or in DGI as identified by function, expression or animal model studies, including *AMELX*, *ENAM*, *KLK4*, *MMP20*, *DSPP*, *SMOC2*, *VPS4B*, and *SSUH2*. However, a novel heterozygous missense mutation (c.1069C > T) was identified in exon 13 of *AMBN* (NM: 016519, chr4:71472172), causing a proline to serine mutation at the conserved amino acid position 357 of the protein (Fig. 4a). The pathogenic variant of *AMBN* was validated by Sanger sequencing followed by co-segregation analysis in all affected and unaffected family members (Fig. 4b). The candidate region was confirmed by additional genotype data from microsatellite markers on 4q, and linkage analysis gave a maximum logarithm of odds score of 4.515 between markers D4S1541 and D4S1558 (Figs. 1 and 4a). Further, this nucleotide change was not detected in DNA samples from 185 normal unrelated controls and 15 dominant AI patients matched for Chinese ethnicity.

### Functional analysis of mutant AMBN

**Fig. 5:**
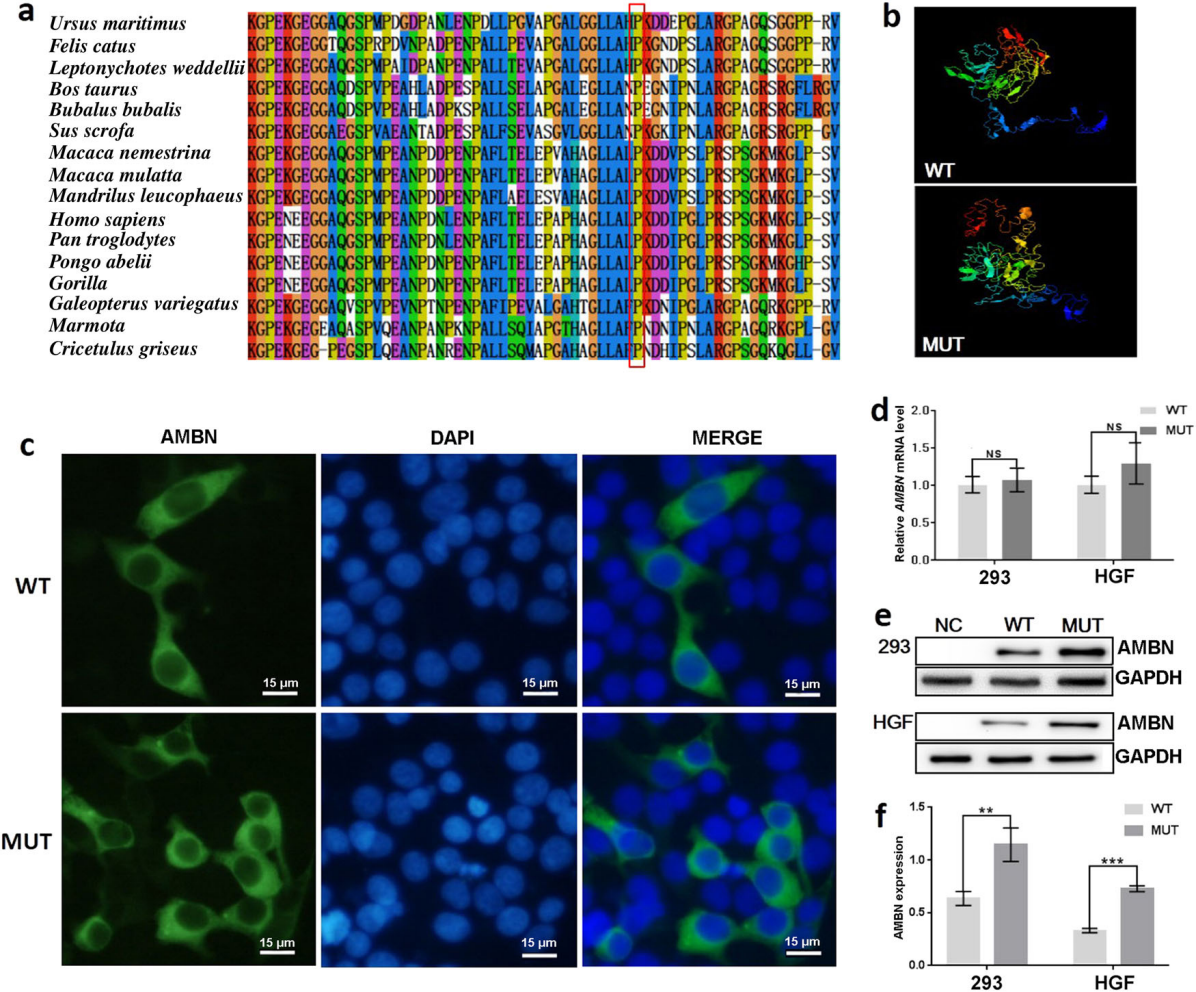
Effect of mutation on AMBN function. **a** Conservation analysis of this abnormal variation by Polyphen-2. The result showed that amino acid 357 of AMBN was highly conserved between different species. **b** The 3D structure of mutated AMBN was different from that of the wild-type predicted by I-TASSER. **c** Subcellular localization of AMBN in HEK293 cells. The mutant AMBN was localized in the cytoplasm similar to the wild-type protein, but the mutant protein exhibited aggregation in transfected HEK293 cells. **d** The mRNA expression level of *AMBN* in HEK293 and HGF cells. Mutant *AMBN* mRNA expression was no different than that of the wild type in either HEK293 cells (*P* > 0.05) or HGF cells (*P* > 0.05). **e** Western blot analysis of AMBN expression. The results showed that mutant AMBN was expressed at a higher level than the normal state. NC, negative control. **f** The protein expression level of AMBN in HEK293 and HGF cells. Compared with the wild type, the protein expression level of AMBN was higher in HEK293 cells (*P* < 0.01) and HGF cells (*P* < 0.001). Data in **d** and **f** are presented as the mean ± SD of three independent experiments. *P < 0.05, **P < 0.01, or ***P < 0.001

The c.1069C > T mutation in *AMBN* changes the three-dimensional (3D) structure of the AMBN protein predicted by I-TASSER, leading to a loss-of-function protein estimated by Polyphen-2 (Fig. 5b). To evaluate the effect of the mutation, the cellular localization of AMBN was analysed using wild type (WT) and mutant constructs in human embryonic kidney (HEK) 293 cells. The result showed that the mutant AMBN was localized in the cytoplasm, which was the same as WT AMBN (Fig. 5c). However, the mutant protein exhibited aggregation in transfected HEK293 cells (Fig. 5c). Moreover, we made two WT and mutant constructs that were transiently expressed in human gingival fibroblasts (HGFs) and HEK293 cells. There was no obvious difference in mRNA expression between cells transfected with the mutated transgenic vector (ΔcAMBN - GFP) or normal transgenic vector (cAMBN - GFP) in either HGF (*P* > 0.05) or HEK293 cells (*P* > 0.05) (Fig. 5d). However, we found that expression of the aberrant protein was significantly increased compared with the WT AMBN protein in both HGF (*P* < 0.001) and HEK293 cells (*P* < 0.01) (Fig. 5e, f).

## Discussion

We have identified a large Chinese pedigree with segregating severe hypoplastic AI and dentin disorders in an autosomal-dominant manner. To predict the affected codon, we used whole exome sequencing and identified a rare variant at exon 13 of the *AMBN* gene on chromosome 4q shared by all affected individuals. Further investigation confirmed that this was a heterozygous missense mutation at position 1069 (c.1069C > T) of the coding region. Human AMBN is composed of 447 residues, many of which are unchanged or only substituted with a similar residue during more than 200 Ma of mammalian evolution.^56^ It is worth noting that the average percentage of proline is 14.09% in the full-length AMBN sequence and 23.78% for the proline-rich region, compared with the 5.1% encountered in most proteins.^56^ Estimation of the codon conservation index along the human AMBN sequence indicated that position P375 of exon 13 is highly conserved.^56^ The evolutionary analyses performed on various secretory calcium-binding phosphoproteins, including AMELX, ENAM, matrix extracellular phosphoglycoprotein, AMTN, and dentin matrix acidic phosphoprotein 1 proteins, which are involved in tooth development, have shown that the patterns of long-term evolutionary conservation have a decisive influence on validating the potential pathogenicity of variants identified in human genetic diseases.^57^ The conserved position sites are very important and such variants usually result in a genetic disorder.^56^ In the present report, our results show that mutation of conserved position P375 of AMBN also causes severe dental defects.

In our study, we report an *AMBN* missense mutation causing severe autosomal-dominant AI. AMBN is an enamel matrix protein (EMP) that could determine the supramolecular enamel matrix assembly by the proline-rich region and is located on chromosome 4 in a region containing the locus for the autosomal hypoplastic form of AI (AIH2).^58^ EMPs consist principally of AMELX, AMBN, and ENAM.^59^ EMPs have a role in maintaining the prismatic structure of growing enamel crystals at the rod and inter-rod boundaries.^60,61^ Heterozygous *Amb*n^+5,6/−5,6^ mice had significantly less well-mineralized prismatic enamel of near-normal thickness compared with WT.^62^ Upon SEM examination, affected teeth exhibited a phenotype in which an ordered thinner prismatic structured layer of enamel was formed with fewer enamel rods and wider inter-rod distances (Fig. 3f). This patient phenotype is similar to that of heterozygous *Ambn*^+5,6 /−5,6^ mice. In addition, the mineral density of enamel was significantly decreased in affected individuals via deletion of AMBN exon 6. The mean mineral density of enamel is approximately 2.47 g/cm^3^. In our study, the mean mineral density of enamel was 2.45 g^.^cm^‒3^ in individuals IV:2 and IV:9, which is consistent with the enamel density values obtained from affected individuals with genomic deletion of AMBN exon 6.

We report the association of a genetic change in *AMBN* with dentin defects in the family identified in this study. In our case, some of the dentin features characteristic of DGI-II and DD-I were observed in the affected individuals (Fig. 2). They appeared similar to those associated with DGI-II, such as bulbous crowns with cervical constriction, mild discoloration, and pulp obliteration. Radiologically, permanent teeth of DD-I have short roots with a crescent-shaped pulpal remnant parallel to the cemento-enamel junction in the permanent dentition and total pulpal obliteration in the dentition.^48,49^ There are usually numerous periapical radiolucencies in non-carious teeth.^48,49^ Compared with DD-I, the clinical crowns of affected teeth in our study had similar pulp and root phenotypes, especially the phenotype of periapical radiolucencies in non-carious teeth (Fig. 2). The clinical phenotypes of teeth from patients in our research ranged from mild to severe with age. Because pulp chamber and canal obliteration are often progressive, radiographic follow-up at various ages is recommended to determine diagnosis.^63^ It has also been reported that *ACP4* mutations caused recessive hypoplastic AI, in which the enamel layer in the affected teeth was thinner and dentin was mildly hypermineralized.^28^ The dentin mineralization in the affected individual was different from most dentin defect cases.^28^ However, in our study, we found that the dentin of teeth of affected individuals was hypomineralized with lower mineral density, which was consistent with most dentin defect cases.

*AMBN* has been reported to play crucial roles in bone development and remodelling/repair.^64^ However, the roles of AMBN in bone are unconfirmed. There were no obvious bone abnormalities identified by detailed clinical evaluations in the family with genomic deletions in *AMBN* exon 6. However, mouse models with loss of *Ambn* function did not have obvious bone defects.^32,65^ In our study, we also did not observe any obvious bone abnormalities in the family, which is consistent with the aforementioned studies.

*AMBN* is believed to participate in ameloblast attachment to the underlying enamel matrix and in modulation of enamel crystal growth.^66^ However, no mutations of *AMBN* have previously been associated with dentin defects. In this report, we found that the genetic change of *AMBN* leads to dentin disorders. Teeth are ectomesenchymal organs that develop via a series of signalling interactions between cells of the oral epithelium and ectomesenchyme.^67^ It has been reported that the *AMBN* gene contains two runt-related transcription factor 2 (*RUNX2*) binding sites, and *AMBN* transcription is regulated by the interaction of the transcription factor *RUNX2* and the *AMBN* promoter.^68,69^ On the other hand, *DSPP* is one of the major pathogenic genes involved in dentin disorders and is also transiently expressed in ameloblasts.^70^ *DSPP* analysis revealed three binding sites for *RUNX2*. *RUNX2* has been shown to be involved in multiple signalling pathways and to control the differentiation of dental pulp cells to odontoblasts during dentinogenesis.^71,72^ *RUNX2* expression in dental pulp cells and odontoblasts was altered in *DSPP* mutants.^73^ Overall, *AMBN*, *RUNX2* and *DSPP* were expressed in ameloblasts and odontoblasts during amelogenesis and dentinogenesis.^38,68,69^ These genes have certain inherent connections that may interact with and restrict each other. Additional mutations in *DSPP* are always associated with dentin defects and have been reported in two families with hypoplastic enamel.^30^ It has been observed that Ambn expression precedes trauma-induced reparative dentin formation in rat molars.^74^ The exposed pulp cavities were effectively closed by thick dentin bridges in recombinant Ambn-treated teeth compared with calcium hydroxide-treated teeth, which were covered only by a thin dentin bridge. This result indicated that *AMBN* plays an important role in the formation of reparative dentin during pulpal healing and links it firmly to odontoblast differentiation and function.^50^ Therefore, we deduced that *AMBN* is involved in odontoblast differentiation and physiology via cell signalling. The interaction between *AMBN*, *RUNX2*, and *DSPP* may underlie the pathogenic mechanism of *AMBN* mutations associated with dentin defects.

ADAI typically affects one or more individuals in each generation of a family.^75^ The clinical manifestations in affected individuals may be consistent or show variable expression, resulting in subtle to substantial differences between all affected individuals in the same family.^76^ The phenotype in ADAI may be predominantly or exclusively hypoplastic, manifesting as thin enamel and spacing between the teeth, crowns reduced in size *ab initio* with thinner enamel, and crowns that are yellowish-brown in hue.^76^ The most abundant enamel protein is amelogenin, which is expressed primarily from a gene on the X-chromosome (*AMELX*), mutations of which have been shown to cause X-linked AI.^77^ *Ambn*^−/−^ mutant mouse models develop autosomal-dominant AI in a dose-dependent manner, which is consistent with the traits of our study of the Chinese kindred, whereas a homozygous 2347 bp genomic deletion and a novel homozygous splice-site mutation (c.532–1G > C) in *AMBN* have been shown to cause autosomal recessive hypoplastic human AI.^8,34^ The enamelin gene, *ENAM*, is implicated in the pathogenesis of the dominant forms of AI.^78^ However, autosomal recessive inheritance has also been recorded for *ENAM* mutations in homozygotes, where heterozygotes presented with a severe and a milder, local form.^18^ The hereditary dentin disorders were documented as autosomal-dominant genetic diseases characterized by abnormal dentin structure affecting either the primary or secondary dentitions.^48,49^

In conclusion, the identification of a heterozygous genomic missense mutation in exon 13 (c.1069C > T) of *AMBN* in a Chinese family confirms, for the first time, that *AMBN* mutations cause autosomal-dominant hypoplastic human AI and dentin defects. This is the third report of an *AMBN* mutation being associated with AI. There is no obvious co-segregating bone or other clinical features associated with this mutation in the affected or unaffected members. In fact, the primary and secondary dentitions are affected by the mutation. The youngest affected individual was entering adulthood when assessed and any effect that the *AMBN* mutation may have on the primary teeth is thus unknown. The mechanisms causing AI and dentin defects in this case are unclear but might be reflected by the nature of the mutation identified, i.e., that this mutation is a missense with a potential gain of function, whereas the mutations previously identified are potentially loss of function. However, further studies reporting on other human patients and *AMBN* variants will be required to confirm this.

## Materials and methods

### Patients and clinical examination

A Han family with hypoplastic AI was identified when the proband, whose teeth had yellowish-brown enamel that broke off in large pieces, came to Nanfang Hospital (Guangdong province, China) for tooth root canal treatment. All the patients in the family received X-rays as part of a clinical examination. All procedures in this study were approved by the institutional review board and ethics committee of Nanfang Hospital, an affiliate of Southern Medical University.

### Tooth ultrastructure analysis

With their informed consent, two wisdom teeth extracted from two patients (IV:2 and IV:9) and a normal control wisdom tooth obtained from the unaffected family member IV:8 were subjected to high-resolution CT using a Skyscan 1176 (Bruker-microCT, Kontich, Belgium) with the following settings: 90 kV, 278 µA, an isotropic resolution of 17.4 µm, and projection rotation steps of 0.50 to 360°. 3D models of the teeth were reconstructed with NRecon v.1.6.3 software (Bruker-microCT). The CT images were calibrated using hydroxyapatite mineral of known densities [0.25 g·cm^‒3^ and 0.75 g·cm^‒3^ (Bruker)]. Measurement of the mean mineral density of the enamel of each tooth was carried out using ImageJ software (http://imagej.nih.gov/ij/). The mean mineral densities of the enamel and the dentin were determined by measuring the mineral density of enamel or dentin on every tenth CT slice taken through each tooth. Calibrated colour contour maps of mineral density were also generated using ImageJ.

For SEM, all extracted teeth were cut into slices using a low-speed hard tissue cutting machine (Buehler, Lake Bluff, IL, USA). The tooth slices were polished using carborundum papers (3 M, Maplewood, MN, USA), followed by a nail buffer. Sections were etched by immersion in 30% phosphoric acid for 30 s, rinsed using excess distilled water for 5 min and dehydrated using ascending concentrations of ethyl alcohol overnight under vacuum. Specimens were fixed and sprayed with gold using an auto sputter coater (Agar Scientific, Elektron Technology, Stansted, UK). The microstructure of the samples was observed with a Hitachi S-3400N scanning electron microscope (Hitachi, Tokyo, Japan) using a 123 eV Nano XFlash R Detector 5010 (Bruker) and an accelerating voltage of 20 kV.

### Whole exome sequencing

Three micrograms of genomic DNA isolated from each of three individuals (IV:1, IV:2, and IV:9) were processed according to the TruSeq DNA Sample Prep Kit protocol (Illumina, San Diego, CA, USA). Agilent SureSelect XT Human All Exon V5 (Agilent Technologies, Santa Clara, CA, USA) was used as the capture reagent. Subsequently, sequencing was performed using a 300 bp paired-end protocol on a Genome Analyzer IIx (Illumina). The read alignment and variant calling were performed by the BaseSpace BWA Enrichment v1.0 App. The sequence was aligned with BWA Genome Alignment Software, and variant calling was performed with GATK using the human reference sequence hg19/GRCh37. Available genomic databases (dbSNP, 1000 Genomes Project, Exome Variant Server, Exome Aggregation Consortium and a local Paris Descartes Bioinformatics platform database) were used to filter exome variants and exclude variants with a frequency > 1%. We performed a number of steps to filter out nucleotide false variants: first, we filed the intronic variants and synonymous single-nucleotide variants; next, we removed the variants annotated in dbSNP without pathogenic relevance to the clinical phenotype using in silico prediction tools. Poor quality variants were excluded (read depth < 30; alternative variant frequency < 5). De novo variants were analysed with PCR and direct Sanger sequencing using DNA from patients and their parents. A total of 185 additional unrelated normal DNA samples and 15 dominant AI samples were used for high-resolution melting analysis and were stored in the Department of Medical Genetics, Southern Medical University, Guangdong, China. All the members participating were recruited after they had provided informed consent in accordance with local ethical approval.

### Short tandem repeat analysis

Seven short tandem repeat (STR) markers (D4S1541, D4S3018, D4S2969, D4S1543, D4S2931, D4S2389, and D4S1558) that flank the *AMBN* gene were selected for STR analysis. The PCR products of these seven loci could be distinguished by co-amplification using fluorescent-labelled STR primers and capillary electrophoresis detection.

### Bioinformatics

To further confirm the function of mutant AMBN, the 3D structures of WT and mutant AMBN were predicted in silico using I-TASSER (Iterative Threading Assembly Refinement, http://zhanglab.ccmb.med.umich.edu/I-TASSER/), and the functional effects of the mutant protein were estimated with Polyphen-2 (Polymorphism Phenotyping v2, http://genetics.bwh.harvard.edu/pph2/).

### Cell transfection and subcellular localization

The coding region of *AMBN* was obtained by synthetic methods, as *AMBN* is not expressed in peripheral blood. The WT coding region was cloned into the Hind III and Bam HI sites of the pcDNA-3.1-flag plasmid (Life Technology, Thermo Fisher Scientific, Waltham, MA, USA) and used as the template for site-directed mutagenesis to clone the mutant form of *AMBN*, ΔcAMBN-pcDNA-3.1-FLAG. HEK293 cells were cultured in 24-well dishes and transfected with WT or mutant *AMBN* plasmids when cells were 50% confluent. Twenty-four hours after transfection, the cells were rinsed three times with phosphate-buffered saline (PBS, Sigma–Aldrich, St Louis, MO, USA) and fixed for 30 min with 4% paraformaldehyde (Sigma–Aldrich). After removing the paraformaldehyde, the dishes were washed with PBS three times, and then, the cells were permeabilized by incubating them in 0.1% Triton X-100 (Thermo Fisher Scientific). After blocking nonspecific reactive sites, the cells were incubated overnight in primary antibody (Sigma–Aldrich; 1:100) in the dark. The next day, the primary antibody was removed and the cells were washed with PBS three times and incubated in a species-specific secondary antibody for 2 h. Nuclei were stained with 4',6-diamidino-2-phenylindole (Sigma–Aldrich) and viewed under a fluorescence microscope (Nikon, Eclipse Ti-U, Tokyo, Japan).

### RNA expression evaluation

HEK293 cells and HGF cells were cultured in Dulbecco's modified Eagle’s medium (DMEM) (Invitrogen, Carlsbad, CA, USA) supplemented with 10% fetal bovine serum (FBS; Gibco, Thermo Fisher Scientific) at 37 °C and 5% CO_2_ in a sterile incubator (Thermo Fisher Scientific). Cells that were 80% confluent were transfected with WT or mutant AMBN recombinant plasmids (ΔcAMBN-pcDNA-3.1-FLAG) using Lipofectamine™ 2000 (Invitrogen) according to the manufacturer’s instructions. Twenty-four hours after transfection, cells were collected and total RNA was isolated. The total RNA was extracted with TRIzol (Thermo Fisher Scientific) and reverse-transcribed to cDNA using the GoScript Reverse Transcription System (Promega, Madison, WI, USA). GoTaqq PCR Master Mix (Promega) was used to measure the mRNA level of WT and mutant *AMBN* alleles. *GAPDH* was used as a reference gene to normalize the expression of the target gene. The transfection step was repeated three times, and all the real-time reverse transcription-PCRs were performed in triplicate to calculate mean values and SDs. The primers for amplifying *AMBN* were as follows: forward: 5′-TTTCTCACGGACCAATGCCA-3′ and reverse: 5′-GCCTCATGCCTCCAAATCCT-3′. Gene expression levels were calculated using the (2^–ΔΔCT^) method. The results were used to calculate the mean values and SD shown in histograms.

### Protein expression

HEK293 cells and HGF cells were cultured in six-well dishes at 37 °C in a 5% CO_2_ atmosphere in a sterile incubator (Thermo Fisher Scientific). DMEM (Invitrogen) supplemented with 10% FBS (GIBCO, Thermo Fisher Scientific) was used for cell culture. To investigate differences in AMBN expression, cells were transfected with 2 μg of WT or mutant *AMBN* recombinant plasmid (ΔcAMBN-FLAG) or empty vector (negative control). After a further 24 h of culture, cells were collected and homogenized in radio-immunoprecipitation assay lysis buffer (Merck-Millipore, Darmstadt, Germany) with the protease inhibitor phenyl methyl sulfonyl fluoride (Beyotime Institute of Biotechnology, Jiangsu, China). Total protein was extracted and assessed using a standard bicinchoninic acid assay (Beyotime). Loading buffer was added before boiling for 5 min. For each sample, 20 μg of protein was loaded onto a 10% SDS-polyacrylamide gel electrophoresis gel (Bio-Rad, Hercules, CA, USA), transferred onto a polyvinylidene difluoride membrane (Millipore), and blocked with 5% skimmed milk (Thermo Fisher Scientific) and 0.1% Tris-buffered saline-Tween 20 (TBST) at room temperature for 1.5 h. The membranes were incubated overnight at 4 °C with mouse anti-FLAG (F1804, Sigma, USA). The membranes were then washed with TBST and incubated with goat anti-mouse IgG (F9006, Sigma) at room temperature for 2 h. SuperSignal West Pico ECL (Thermo Fisher Scientific) was freshly prepared just before detection of proteins. Protein expression was detected by exposure to an automatic chemiluminescence image analysis system (Tanon, Shanghai, China) and evaluated using SuperSignal™ West Femto Maximum Sensitivity Substrate (Thermo Fisher Scientific).

### Statistical analyses

The significance of differences between two groups was determined using Student’s *t*-test followed by Bernoulli correction and adjusted *P*-values were used to identify significant differences. The quantified results are shown as the mean ± SD. *P* < 0.05 was considered statistically significant, *P < 0.05, **P < 0.01, or ***P < 0.001.

## Electronic supplementary material

3D reconstruction vedio of normal tooth

3D reconstruction vedio of IV-2

3D reconstruction vedio of IV-9
